# National, State, and Selected Local Area Vaccination Coverage Among Children Aged 19–35 Months — United States, 2013

**Published:** 2014-08-29

**Authors:** Laurie D. Elam-Evans, David Yankey, James A. Singleton, Maureen Kolasa

**Affiliations:** 1 Immunization Services Division, National Center for Immunization and Respiratory Diseases, CDC

In the United States, among children born during 1994–2013, vaccination will prevent an estimated 322 million illnesses, 21 million hospitalizations, and 732,000 deaths during their lifetimes (1). Since 1994, the National Immunization Survey (NIS) has monitored vaccination coverage among children aged 19–35 months in the United States. This report describes national, regional, state, and selected local area vaccination coverage estimates for children born January 2010–May 2012, based on results from the 2013 NIS. In 2013, vaccination coverage achieved the 90% national *Healthy People 2020* target* for ≥1 dose of measles, mumps, and rubella vaccine (MMR) (91.9%); ≥3 doses of hepatitis B vaccine (HepB) (90.8%); ≥3 doses of poliovirus vaccine (92.7%); and ≥1 dose of varicella vaccine (91.2%). Coverage was below the *Healthy People 2020* targets for ≥4 doses of diphtheria, tetanus, and pertussis vaccine (DTaP) (83.1%; target 90%); ≥4 doses of pneumococcal conjugate vaccine (PCV) (82.0%; target 90%); the full series of *Haemophilus influenzae* type b vaccine (Hib) (82.0%; target 90%); ≥2 doses of hepatitis A vaccine (HepA) (54.7%; target 85%); rotavirus vaccine (72.6%; target 80%); and the HepB birth dose (74.2%; target 85%).† Coverage remained stable relative to 2012 for all of the vaccinations with *Healthy People 2020* objectives except for increases in the HepB birth dose (by 2.6 percentage points) and rotavirus vaccination (by 4.0 percentage points). The percentage of children who received no vaccinations remained below 1.0% (0.7%). Children living below the federal poverty level had lower vaccination coverage compared with children living at or above the poverty level for many vaccines, with the largest disparities for ≥4 doses of DTaP (by 8.2 percentage points), full series of Hib (by 9.5 percentage points), ≥4 doses of PCV (by 11.6 percentage points), and rotavirus (by 12.6 percentage points). MMR coverage was below 90% for 17 states. Reaching and maintaining high coverage across states and socioeconomic groups is needed to prevent resurgence of vaccine-preventable diseases.

NIS is a random-digit–dialed cellular§ and landline telephone survey of households with children aged 19–35 months in the 50 states, the District of Columbia, selected local areas, Guam, and the U.S. Virgin Islands (USVI).¶ These household interviews are followed by a survey mailed to the child’s vaccination providers (with consent of the respondent) to obtain provider-confirmed vaccination histories. Data are weighted to be representative of the population of children aged 19–35 months, and are adjusted for multiple phone lines, mixed telephone use (i.e. landline and cellular), household nonresponse, and the exclusion of phoneless households. Details regarding NIS methodology, including methods for synthesizing provider-reported immunization histories and weighting, have been described previously.** The sample size of children with adequate provider data used for national estimates was 13,611, with an additional 449 children from USVI and Guam.†† For completed interviews (excluding Guam and USVI), 3,152 by landline (63.5%) and 10,459 by cell phone (59.8%) had adequate vaccination data. The national Council of American Survey Research Organization (CASRO) response rates were 62.3% for landline and 30.5% for cell phone frames.§§ Coverage estimates for Hib¶¶ and rotavirus*** vaccines take into account the type of vaccine used because the number of doses required depends on the manufacturer. Logistic regression was used to examine differences among racial and ethnic populations, controlling for poverty status. Statistical analyses were conducted using t-tests, based on weighted data and accounting for the complex survey design. A p-value of <0.05 was considered statistically significant.

## National Vaccination Coverage

In 2013, national vaccination coverage among children aged 19–35 months was 83.1% for ≥4 DTaP doses, 92.7% for ≥3 poliovirus doses, 91.9% for ≥1 MMR dose, 82.0% for the full series of Hib, 90.8% for ≥3 HepB doses, 91.2% for ≥1 varicella dose, and 82.0% for ≥4 PCV doses (Table 1). Coverage remained stable for these vaccinations relative to 2012. Coverage with the combined vaccine series††† of these vaccines was 70.4%, similar to coverage in 2012. Coverage increased from 2012 to 2013 for HepB (birth dose) (from 71.6% to 74.2%), for rotavirus vaccine (from 68.6% to 72.6%), and for ≥1 dose of HepA (from 81.5% to 83.1%). No change was observed in the percentage of children who received no vaccinations.

## Vaccination Coverage by Selected Demographic Characteristics

Children living below the poverty level§§§ had lower coverage than children living at or above the poverty level for several vaccines, including ≥3 and ≥4 DTaP doses, ≥3 poliovirus doses, Hib (full series), ≥3 HepB doses, ≥3 and ≥4 PCV doses, rotavirus, and the combined vaccine series (Table 2). However, children living below the poverty level had higher coverage than children living at or above the poverty level for HepB (birth dose).

In 2013, black children¶¶¶ had lower coverage compared with white children for ≥3 and ≥4 DTaP doses, Hib (full series), ≥4 PCV doses, rotavirus, and the combined vaccine series (Table 2). After adjustment for poverty status, these disparities were reduced but remained statistically significant, except for the combined vaccine series. Conversely, other groups had higher coverage for various vaccines compared with white children. American Indian/Alaska Native (AI/AN) and Asian children had higher coverage than white children for ≥1 MMR dose and ≥1 varicella dose. AI/AN children also had higher coverage than white children for ≥3 HepB doses, and Asian children had higher coverage than white children for ≥2 HepA doses. Black and Hispanic children had higher coverage than white children for HepB (birth dose).

## Vaccination Coverage by State

In 2013, wide geographic variation in vaccination coverage was observed among the states (Table 3). Coverage for ≥1 MMR dose ranged from 86.0% (Colorado, Ohio, and West Virginia) to 96.3% (New Hampshire). Coverage ranged from 74.3% (Arkansas) to 93.3% (Massachusetts) for ≥4 DTaP doses, from 44.8% (Vermont) to 88.0% (Kentucky) for HepB (birth dose), from 33.6% (Wyoming) to 72.1% (Connecticut) for ≥2 HepA doses, from 56.0% (Arkansas) to 84.4% (Rhode Island) for rotavirus, and from 57.1% (Arkansas) to 82.1% (Rhode Island) for the combined vaccine series.

### Discussion

The results of the 2013 NIS indicate that vaccination coverage among children aged 19–35 months increased relative to 2012 NIS estimates for some vaccines (rotavirus, HepB birth dose, and ≥1 HepA dose) and remained stable for the others, and less than 1% of children had not received any vaccinations. The national *Healthy People 2020* targets were met in 2013 for four vaccines (≥1 MMR, ≥3 HepB, ≥3 poliovirus, and ≥1 varicella doses). Additionally, four vaccines were within eight percentage points of their *Healthy People 2020* targets (≥4 DTaP doses, the full series of Hib, ≥4 PCV doses, and rotavirus), but coverage increased from 2012 to 2013 only for rotavirus vaccination. Further, disparities in coverage by poverty level were larger for these four vaccines compared with vaccines meeting their *Healthy People 2020* targets. Although coverage with ≥2 HepA doses was 30 percentage points below the 85% 2020 target and did not increase from 2012 to 2013, ≥1 HepA dose coverage increased slightly and reached 83% in 2013.

In 2012 and 2013, coverage for DTaP, PCV, and the full series of Hib remained at similar levels (81%–83%). These vaccines require a booster dose during the second year of life, when the opportunities for catch-up doses with these vaccines are fewer because of declining frequency of well-child visits. CDC recommends the use of clinician and system-based interventions to increase opportunities for vaccination, including use of immunization information systems (IIS), clinician assessment and feedback, clinician reminders, and standing orders (2).

DTaP, PCV, and Hib coverage were 8 to 12 percentage points lower for children living below the poverty level compared with children living at or above the poverty level. Parents and caregivers of children living below poverty might face additional challenges in maintaining well-child visits and thus be more likely to fall behind on booster doses. Children living below poverty also had rotavirus coverage that was 13 percentage points lower than that of children living at or above the poverty level. The first dose of rotavirus vaccine should be given before age 14 weeks and 6 days, and the final dose should be given by 8 months (3). Children living below poverty might be more likely to miss these milestones and thus not able to start or complete the series. The Vaccines for Children program likely has been successful in reducing differences in vaccination coverage between children living at or above poverty level compared with those below the poverty level for these vaccines and in removing poverty differences for vaccines such as MMR and varicella (1). To further reduce disparities, clinician and system-based interventions should be targeted to communities with a high proportion of the population living below the poverty level. Interventions to improve parental knowledge about vaccines and to further facilitate access to vaccinations can also help to reduce disparities in coverage.

Despite a national MMR vaccination coverage level of 91.9%, one child in 12 in the United States is not receiving their first dose of MMR vaccine on time, underscoring considerable measles susceptibility across the country. Vaccination coverage continued to vary by state. In 2013, there were 10 states with ≥1 MMR dose coverage levels ≥95%, and 17 states with ≥1 MMR dose coverage below the *Healthy People 2020* target of 90%. Through August 8, 2014, a total of 593 measles cases had been reported from 21 states, the highest number reported in the United States since measles was declared eliminated in the United States in 2000; most cases have occurred in persons who were unvaccinated or had unknown vaccination status; updated provisional case counts are available at http://www.cdc.gov/measles/index.html. Given the large number of cases this year and the continuing risk for importation, clinicians should have a heightened awareness of the potential for measles in their communities and the importance of vaccination to prevent measles. Communities with lower MMR coverage are more vulnerable to measles transmission. Outbreaks of measles most commonly occur in communities with pockets of persons who were unvaccinated because of philosophic or religious beliefs (4). Pockets of unvaccinated persons also occur in states with high vaccination coverage, highlighting the importance of state health departments assessing measles susceptibility at the local level.

State and local health departments can identify communities with lower MMR and other vaccination rates among children using IIS (5). Based on 2012 reports from 54 of 56 state and local immunization awardees, 86% of U.S. children aged <6 years participated in IIS (5), which are effective in increasing vaccination rates through their capabilities for 1) generating patient reminder and recall notifications, enabling clinician assessment and feedback, and providing clinician reminders; 2) determining patient vaccination status for decisions made by clinicians, health departments, and schools; 3) guiding public health responses to outbreaks of vaccine-preventable disease; 4) informing assessments of vaccination coverage by examining missed vaccination opportunities and disparities in vaccination coverage; and 5) facilitating vaccine management and accountability (2). The full potential of IIS can be achieved by meeting or exceeding new functional standards for IIS developed by CDC for 2013–2017 and fully utilizing IIS for program planning, implementation, and evaluation (5). In addition to IIS, other sources of information on local coverage that might be available include school or community level data from monitoring school vaccination requirements (6) and county level estimates from NIS (7). Taken together, local coverage estimates from IIS and other sources can provide critical data to inform programs and interventions at the county level that might subsequently further increase vaccination coverage.

The findings in this report are subject to at least three limitations. First, the household response rates for landline and cell phone samples were 62.3% and 30.5%, respectively. Furthermore, only 63.5% of landline and 59.8% of cell phone completed interviews had adequate vaccination data. Thus, estimates might have been biased, even after sample weights were adjusted to combine landline and cell samples and adjusted to correct for nonresponse, exclusion of households without telephones, and overlapping samples of mixed (landline and cell) telephone users. Results are weighted to key population controls. Although weighting does not guarantee against bias, it does mitigate and minimize the bias. Second, although response rates are within 1–3 percentage points of previous year and weights have been adjusted to reflect the increasing prevalence of cell-only households over time, nonresponse bias might have changed over time, which could affect interpretation of comparisons across data years. Analyses of total survey error for the NIS for 2010,**** 2011 and 2012 (through June) indicated bias in estimates attributable to incomplete sample frame and selection bias was low, on the order of less than two percentage points (8). Future analyses will quantify the amount of bias that might be occurring in later years of NIS data. Third, NIS estimates of ≥2 HepA doses might underestimate coverage of children before age 3 years. The first dose of HepA is recommended during age 12–23 months, and the second dose is recommended at 6–18 months after the first dose (3). Children’s vaccination status in NIS is determined up to age 19–35 months, so some children might have received their second dose, or be due to receive their second dose, after the survey was conducted.

What is already known on this topic?

*Healthy People 2020* has set childhood vaccination targets of 90% for ≥1 dose measles, mumps, and rubella vaccine, ≥3 doses of hepatitis B vaccine, ≥3 doses of poliovirus vaccine, ≥1 dose of varicella vaccine, ≥4 doses of diphtheria, tetanus, and pertussis vaccine, ≥4 doses of pneumococcal conjugate vaccine, and the full series of *Haemophilus influenzae* type b vaccine. For these and other vaccines, the National Immunization Survey estimates coverage among U.S. children aged 19–35 months.

What is added by this report?

In 2013, childhood vaccination coverage remains near or above national target levels for ≥1 dose of measles, mumps, and rubella vaccine (91.9%), ≥3 doses of hepatitis B vaccine (90.8%), ≥3 doses of poliovirus vaccine (92.7%), and ≥1 dose of varicella vaccine (91.2%); however, coverage varied by state, and differences in coverage by income persist.

What are the implications for public health practice?

To sustain high coverage and improve coverage for more recently recommended vaccines and those that require booster doses after age 12 months, efforts are needed by parents, clinicians, health systems, and local and state health departments to implement interventions recommended by the *Guide to Community Preventive Services*. Further development and use of immunization information systems by state and local health departments can further identify local pockets of undervaccinated children to ensure that all children remain adequately protected.

Coverage for many childhood vaccinations during 1994–2013 at, near, or above 90% has contributed to low levels of most vaccine-preventable diseases and estimated net savings of $1.38 trillion in total societal costs over the lifetimes of children born during that period (1). Results of the 2013 NIS indicate sustained high vaccination coverage and low proportion of children aged 19–35 months who have not received any vaccinations. Established in 1994 and reaching its 20th year in 2013, the NIS will continue to monitor coverage levels overall and in subpopulations (e.g., by poverty status, race/ethnicity, state, and selected local areas) to identify gaps in vaccination coverage. Further development and use of IIS by state and local health departments can further identify local pockets of undervaccinated children to ensure that all children remain adequately protected. To sustain high coverage and improve coverage for more recently recommended vaccines and those that require booster doses after age 12 months, efforts are needed by parents, clinicians, health systems, and local and state health departments to implement the interventions recommended by the *Guide to Community Preventive Services* (2). In addition to use of IIS, these interventions are aimed at increasing community demand for vaccination, enhancing access to health services, and implementing provider- and system-based interventions.

## Figures and Tables

**TABLE 1:** Estimated vaccination coverage among children aged 19–35 months, by selected vaccines and dosages — National Immunization Survey, United States, 2009–2013*

	2009		2010		2011		2012		2013	
					
Vaccine and dosage	%	(95% CI)	%	(95% CI)	%	(95% CI)	%	(95% CI)	%	(95% CI)
**DTaP**										
≥3 doses	95.0	(±0.6)	95.0	(±0.6)	95.5	(±0.5)	94.3	(±0.7)	94.1	(±0.9)
≥4 doses	83.9	(±1.0)	84.4	(±1.0)	84.6	(±1.0)	82.5	(±1.2)	83.1	(±1.3)
**Poliovirus (≥3 doses)**	92.8	(±0.7)	93.3	(±0.7)	93.9	(±0.6)	92.8	(±0.7)	92.7	(±1.0)
**MMR (≥1 dose)**	90.0	(±0.8)	91.5	(±0.7)	91.6	(±0.8)	90.8	(±0.8)	91.9	(±0.9)
**Hib** †										
Primary series	92.1	(±0.8)	92.2	(±0.8)	94.2	(±0.6)	93.3	(±0.7)	93.7	(±0.9)
Full series	54.8	(±1.4)	66.8	(±1.3)	80.4	(±1.1)	80.9	(±1.2)	82.0	(±1.3)
**HepB**										
≥3 doses	92.4	(±0.7)	91.8	(±0.7)	91.1	(±0.7)	89.7	(±0.9)	90.8	(±1.0)
1 dose by 3 days (birth)§	60.8	(±1.3)	64.1	(±1.3)	68.6	(±1.3)	71.6	(±1.4)	74.2	(±1.4)¶
**Varicella (≥1 dose)**	89.6	(±0.8)	90.4	(±0.8)	90.8	(±0.7)	90.2	(±0.8)	91.2	(±0.9)
**PCV**										
≥3 doses	92.6	(±0.7)	92.6	(±0.8)	93.6	(±0.6)	92.3	(±0.8)	92.4	(±1.0)
≥4 doses	80.4	(±1.2)	83.3	(±1.0)	84.4	(±1.0)	81.9	(±1.1)	82.0	(±1.3)
**HepA**										
≥1 dose	75.0	(±1.1)	78.3	(±1.1)	81.2	(±1.0)	81.5	(±1.1)	83.1	(±1.2)¶
≥2 doses	46.6	(±1.4)	49.7	(±1.4)	52.2	(±1.4)	53.0	(±1.5)	54.7	(±1.6)
**Rotavirus** **	43.9	(±1.4)	59.2	(±1.4)	67.3	(±1.3)	68.6	(±1.4)	72.6	(±1.5)¶
**Combined series** ††	44.3	(±1.4)	56.6	(±1.3)	68.5	(±1.3)	68.4	(±1.4)	70.4	(±1.5)
**Children who received no vaccinations**	0.6	(±0.1)	0.7	(±0.2)	0.8	(±0.2)	0.8	(±0.1)	0.7	(±0.3)

**Abbreviations:** CI = confidence interval; DTaP = diphtheria, tetanus toxoids, and acellular pertussis vaccine (includes children who might have been vaccinated with diphtheria and tetanus toxoids vaccine, or diphtheria, tetanus toxoids, and pertussis vaccine); MMR = measles, mumps, and rubella vaccine; Hib = *Haemophilus influenzae* type b vaccine; HepB = hepatitis B vaccine; PCV = pneumococcal conjugate vaccine; HepA = hepatitis A vaccine.

* For 2009, includes children born January 2006-July 2008; for 2010, children born January 2007-July 2009; for 2011, children born January 2008-May 2010; for 2012, children born January 2009-May 2011; and for 2013, children born January 2010-May 2012.

† Hib primary series: receipt of ≥2 or ≥3 doses, depending on product type received. Full series: receipt of ≥3 or ≥4 doses, depending on product type received (primary series and booster dose). Hib coverage for primary or full series not available until 2009.

§ HepB administered from birth through age 3 days.

¶ Statistically significant change in coverage compared with 2012 (p<0.05).

** Rotavirus vaccine includes ≥2 or ≥3 doses, depending on the product type received (≥2 doses for Rotarix [RV1] or ≥3 doses for RotaTeq [RV5]).

†† The combined (4:3:1:3*:3:1:4) vaccine series includes ≥4 doses of DTaP, ≥3 doses of poliovirus vaccine, ≥1 dose of measles-containing vaccine, full series of Hib vaccine (≥3 or ≥4 doses, depending on product type), ≥3 doses of HepB, ≥1 dose of varicella vaccine, and ≥4 doses of PCV.

**TABLE 2:** Estimated vaccination coverage among children aged 19–35 months, by selected vaccines and dosages, race/ethnicity,* and poverty level† — National Immunization Survey, United States, 2013§

	Race/Ethnicity														Poverty level			
		
	White, non-Hispanic		Black, non-Hispanic		Hispanic		American Indian/Alaska Native only, non-Hispanic		Asian, non-Hispanic		Native Hawaiian or other Pacific Islander, non-Hispanic		Multiracial, non-Hispanic		At or Above		Below	
									
Vaccine and dosage	%	(95% CI)	%	(95% CI)	%	(95% CI)	%	(95% CI)	%	(95% CI)	%	(95% CI)	%	(95% CI)	%	(95% CI)	%	(95% CI)
**DTaP**																		
≥3 doses	95.1	(±0.9)	92.4	(±2.3)¶	93.4	(±2.4)	92.4	(±6.4)	96.0	(±4.6)	NA	(±NA)	92.4	(±3.6)	95.6	(±0.8)	91.2	(±2.1)**
≥4 doses	85.3	(±1.4)	74.7	(±4.2)¶	82.3	(±3.2)	78.1	(±8.8)	89.0	(±5.2)	NA	(±NA)	83.1	(±4.5)	86.0	(±1.3)	77.8	(±2.7)**
**Poliovirus (≥3 doses)**	93.7	(±1.0)	91.2	(±2.6)	91.6	(±2.7)	92.2	(±6.4)	95.5	(±4.7)	NA	(±NA)	90.8	(±3.7)	94.4	(±0.8)	89.2	(±2.4)**
**MMR (≥1 dose)**	91.5	(±1.1)	90.9	(±2.5)	92.1	(±2.5)	96.3	(±2.8)¶	96.7	(±1.7)¶	90.4	(±9.7)	91.5	(±3.1)	92.5	(±0.9)	90.5	(±2.1)
**Hib** ††																		
≥3 doses	93.7	(±1.0)	90.7	(±2.5)¶	92.7	(±2.5)	89.5	(±6.8)	92.9	(±4.9)	90.5	(±9.6)	91.4	(±3.7)	94.6	(±0.8)	89.6	(±2.2)**
Primary series	94.6	(±0.8)	91.4	(±2.4)¶	93.3	(±2.4)	94.3	(±6.1)	93.8	(±4.8)	90.5	(±9.6)	92.3	(±3.6)	95.1	(±0.8)	91.0	(±2.0)**
Full series	84.2	(±1.4)	74.9	(±4.2)¶	80.9	(±3.3)	82.9	(±7.8)	82.0	(±6.2)	NA	(±NA)	84.9	(±4.1)	85.3	(±1.4)	75.8	(±2.8)**
**HepB**																		
≥3 doses	91.0	(±1.0)	91.1	(±2.4)	89.7	(±2.6)	96.1	(±4.3)¶	92.0	(±5.1)	94.9	(±5.6)	90.7	(±3.5)	92.0	(±0.9)	88.3	(±2.2)**
1 dose by 3 days (birth)§§	71.9	(±1.8)	76.7	(±3.7)¶	77.8	(±3.5)¶	NA	(±NA)	73.7	(±6.5)	NA	(±NA)	72.3	(±5.9)	72.1	(±1.7)	78.3	(±2.7)**
**Varicella (≥1 dose)**	90.0	(±1.2)	92.1	(±2.2)	92.0	(±2.5)	95.4	(±3.1)¶	96.0	(±2.0)¶	88.7	(±9.2)	91.0	(±3.0)	91.6	(±0.9)	90.3	(±2.1)
**PCV**																		
≥3 doses	93.1	(±1.0)	90.8	(±2.6)	92.2	(±2.5)	92.3	(±6.1)	92.0	(±4.9)	90.9	(±8.6)	91.5	(±3.6)	94.2	(±0.8)	88.8	(±2.3)**
≥4 doses	84.1	(±1.5)	76.1	(±3.8)¶	80.4	(±3.4)	79.0	(±8.3)	85.6	(±5.4)	NA	(±NA)	83.0	(±4.4)	86.1	(±1.4)	74.5	(±2.7)**
**HepA (≥2 doses)**	53.4	(±1.9)	49.1	(±4.3)	56.6	(±4.0)	NA	(±NA)	67.3	(±6.8)¶	NA	(±NA)	57.8	(±6.0)	56.1	(±1.9)	53.5	(±2.9)
**Rotavirus** ¶¶	74.8	(±1.7)	62.1	(±4.3)¶	73.7	(±3.5)	NA	(±NA)	74.9	(±6.7)	NA	(±NA)	72.8	(±5.3)	76.9	(±1.6)	64.3	(±2.9)**
**Combined series** ***	72.1	(±1.8)	65.0	(±4.4)¶	69.3	(±3.8)	70.1	(±9.2)	72.7	(±6.6)	NA	(±NA)	71.8	(±5.2)	73.8	(±1.7)	64.4	(±3.0)**

**Abbreviations:** CI = confidence interval; DTaP = diphtheria, tetanus toxoids, and acellular pertussis vaccine (includes children who might have been vaccinated with diphtheria and tetanus toxoids vaccine, or diphtheria, tetanus toxoids, and pertussis vaccine); NA = not available (estimate not available if the unweighted sample size for the denominator was <30 or 95% CI half width/estimate >0.588 or 95% CI half width was ≥10); MMR = measles, mumps, and rubella vaccine; Hib = *Haemophilus influenzae* type b vaccine; HepB = hepatitis B vaccine; PCV = pneumococcal conjugate vaccine; HepA = hepatitis A vaccine.

* Children’s race/ethnicity was reported by parent or guardian. Children identified in this report as white, black, Asian, American Indian/Alaska Native, Native Hawaiian or other Pacific Islander, or multiracial were reported by the parent or guardian as non-Hispanic. Children identified as multiracial had more than one race category selected. Children identified as Hispanic might be of any race.

† Children were classified as below poverty if their total family income was less than the poverty threshold specified for the applicable family size and number of children aged <18 years. Children with total family income at or above the poverty threshold specified for the applicable family size and number of children aged <18 years were classified as at or above poverty. A total of 535 children with adequate provider data and missing data on income were excluded from the analysis. Poverty thresholds reflect yearly changes in the Consumer Price Index. Additional information available at http://www.census.gov/hhes/www/poverty.html.

§ Children in the 2013 National Immunization Survey were born January 2010-May 2012.

¶ Statistically significant difference (p<0.05) in estimated vaccination coverage by race/ethnicity. Children identified as non-Hispanic white were the reference group.

** Statistically significant difference (p<0.05) in estimated vaccination coverage by poverty level. Children living at or above poverty were the reference group.

†† Hib primary series: receipt of ≥2 or ≥3 doses, depending on product type received; full series: primary series and booster dose includes receipt of ≥3 or ≥4 doses, depending on product type received.

§§ HepB administered from birth through age 3 days.

¶¶ Includes ≥2 or ≥3 doses, depending on product type received (≥2 doses for Rotarix [RV1] or ≥3 doses for RotaTeq [RV5]).

*** The combined (4:3:1:3*:3:1:4) vaccine series includes ≥4 doses of DTaP, ≥3 doses of poliovirus vaccine, ≥1 dose of measles-containing vaccine, full series of Hib vaccine (≥3 or ≥4 doses, depending on type), ≥3 doses of HepB, ≥1 dose of varicella vaccine, and ≥4 doses of PCV.

**TABLE 3:** Estimated vaccination coverage with selected individual vaccines and a combined vaccine series* among children aged 19–35 months, by U.S. Department of Health and Human Services (HHS) region and state and local area — National Immunization Survey, United States, 2013†

HHS region, state and local area	MMR (≥1 dose)		DTaP (≥4 doses)		Hep B (birth)§		HepA (≥2 doses)		Rotavirus¶		Combined vaccine series*	
					
	%	(95% CI)	%	(95% CI)	%	(95% CI)	%	(95% CI)	%	(95% CI)	%	(95% CI)
**United States overall**	**91.9**	**(±0.9)**	**83.1**	**(±1.3)**	**74.2**	**(±1.4)** **	**54.7**	**(±1.6)**	**72.6**	**(±1.5)** **	**70.4**	**(±1.5)**
**HHS Region I**	**94.2**	**(±2.2)**	**90.9**	**(±2.5)**	**74.6**	**(±3.7)**	**63.2**	**(±4.4)**	**81.4**	**(±3.5)**	**77.1**	**(±3.7)**
Connecticut	91.4	(±5.4)	88.0	(±5.9)	75.2	(±7.5)	72.1	(±7.5)	81.1	(±6.3)	78.2	(±6.8)
Maine	91.0	(±4.5)	87.9	(±5.7)	68.9	(±7.4)	57.4	(±7.7)	72.0	(±7.1)	68.0	(±7.5)
Massachusetts	95.8	(±3.6)	93.3	(±4.0)	78.0	(±6.4)	62.7	(±8.0)	84.0	(±6.3)	78.5	(±6.6)
New Hampshire	96.3	(±2.6)	91.3	(±3.9)	74.1	(±6.5)	53.3	(±7.7)	78.2	(±6.7)	74.9	(±6.8)
Rhode Island	95.6	(±3.3)	91.6	(±4.9)	72.7	(±7.0)	60.9	(±8.2)	84.4	(±6.2)	82.1	(±6.7)**
Vermont	91.2	(±4.0)	85.8	(±5.1)	44.8	(±6.8)	48.5	(±6.8)**	73.4	(±6.1)**	66.9	(±6.6)
**HHS Region II**	**95.5**	**(±1.9)** **	**86.5**	**(±3.1)**	**62.5**	**(±4.2)**	**49.3**	**(±4.4)**	**72.3**	**(±4.0)** **	**72.4**	**(±4.1)** **
New Jersey	95.6	(±3.3)	86.4	(±5.3)	59.8	(±7.2)	51.2	(±7.4)	69.0	(±6.9)	72.9	(±6.8)
New York	95.5	(±2.3)**	86.6	(±3.8)	63.7	(±5.2)	48.4	(±5.5)	73.8	(±4.8)**	72.2	(±5.0)**
City of New York	96.8	(±2.5)**	86.0	(±5.3)	61.2	(±7.1)	49.4	(±7.3)	67.0	(±7.1)**	69.8	(±6.9)
Rest of state	94.2	(±3.9)	87.2	(±5.5)	66.3	(±7.6)	47.3	(±8.2)	80.7	(±6.4)	74.6	(±7.4)**
**HHS Region III**	**92.1**	**(±2.6)**	**85.2**	**(±3.4)**	**77.9**	**(±3.8)**	**55.1**	**(±4.3)**	**77.8**	**(±3.7)** **	**73.1**	**(±4.0)**
Delaware	94.8	(±3.4)	87.9	(±5.0)	83.6	(±5.3)**	64.2	(±7.0)	83.9	(±5.6)	71.8	(±6.6)
District of Columbia	96.2	(±3.1)	86.2	(±5.8)	78.3	(±6.9)	66.2	(±8.4)	68.4	(±8.1)**	76.9	(±7.2)
Maryland	95.3	(±4.4)	87.4	(±6.5)	75.4	(±7.7)	55.6	(±9.2)	83.7	(±6.6)**	75.8	(±8.0)
Pennsylvania	93.3	(±3.2)**	88.7	(±3.9)**	83.3	(±4.3)	58.3	(±5.8)	77.2	(±5.3)	75.5	(±5.2)
Philadelphia	95.9	(±2.7)	88.7	(±4.5)	77.9	(±5.9)	59.5	(±7.2)	73.4	(±6.4)	76.7	(±6.4)
Rest of state	92.8	(±3.8)**	88.7	(±4.5)**	84.4	(±5.0)	58.1	(±6.7)	78.0	(±6.2)	75.3	(±6.1)
Virginia	88.6	(±7.0)	78.8	(±9.3)	72.3	(±10.2)	48.0	(±10.8)	76.2	(±9.2)	69.2	(±10.0)
West Virginia	86.0	(±5.8)	83.4	(±6.2)	73.9	(±7.9)	57.5	(±8.4)	68.4	(±7.8)	65.5	(±7.9)
**HHS Region IV**	**93.0**	**(±1.7)**	**82.8**	**(±3.0)**	**73.5**	**(±3.3)**	**51.3**	**(±3.4)**	**68.9**	**(±3.4)**	**70.8**	**(±3.5)**
Alabama	89.7	(±5.8)	84.0	(±7.3)	81.7	(±7.1)	59.2	(±8.9)	74.8	(±7.8)	77.0	(±7.8)
Florida	93.4	(±4.0)	80.3	(±7.7)	58.0	(±8.3)	48.7	(±8.0)	66.0	(±8.1)	70.0	(±8.7)
Georgia	93.9	(±4.1)	83.5	(±7.9)	76.4	(±8.8)**	58.0	(±10.1)	64.6	(±10.2)	69.8	(±9.8)
Kentucky	89.5	(±5.1)	84.1	(±6.4)	88.0	(±5.6)	41.4	(±8.6)	66.4	(±8.5)	72.7	(±8.0)
Mississippi	95.2	(±3.0)	87.4	(±5.4)	79.2	(±7.1)	39.1	(±8.8)	63.2	(±8.6)	74.6	(±7.7)
North Carolina	96.0	(±3.3)**	87.5	(±5.3)	82.1	(±6.1)	51.6	(±7.7)	75.4	(±7.2)	72.0	(±7.5)
South Carolina	89.2	(±5.3)	77.3	(±7.5)	76.1	(±7.4)	52.5	(±8.8)	69.9	(±8.2)	66.5	(±8.3)
Tennessee	92.3	(±4.4)	81.1	(±6.0)	76.6	(±5.8)	52.6	(±7.1)	73.3	(±7.2)	68.5	(±6.8)
**HHS Region V**	**90.1**	**(±1.9)**	**81.6**	**(±2.5)**	**76.5**	**(±2.6)**	**53.0**	**(±3.0)**	**70.9**	**(±2.8)**	**68.0**	**(±2.9)**
Illinois	91.4	(±3.1)	82.7	(±4.5)	71.4	(±5.1)	48.4	(±5.5)	72.6	(±5.0)	66.8	(±5.3)
City of Chicago	90.0	(±5.2)	82.0	(±7.3)	78.9	(±8.2)	43.6	(±9.3)	76.1	(±7.7)	64.4	(±8.5)
Rest of state	91.9	(±3.8)	83.0	(±5.5)	68.7	(±6.2)	50.1	(±6.7)	71.4	(±6.2)	67.7	(±6.5)
Indiana	92.0	(±3.6)	82.1	(±5.3)	82.8	(±5.7)	61.0	(±6.9)**	65.7	(±7.1)	68.5	(±6.7)
Michigan	89.2	(±5.1)	79.6	(±6.6)	82.5	(±6.1)	51.2	(±7.9)	70.1	(±7.2)	70.0	(±7.4)
Minnesota	90.8	(±5.5)	90.5	(±5.0)	63.8	(±8.6)	54.3	(±9.1)	80.3	(±6.9)	74.1	(±7.8)
Ohio	86.0	(±5.2)	75.8	(±7.0)	78.1	(±6.4)	49.2	(±7.6)	66.5	(±7.5)	61.7	(±7.5)
Wisconsin	93.2	(±4.2)	84.0	(±6.1)	80.5	(±6.0)	63.2	(±7.5)	73.6	(±6.9)	72.8	(±7.1)
**HHS Region VI**	**91.5**	**(±2.1)**	**80.4**	**(±3.2)**	**80.5**	**(±2.8)** **	**58.9**	**(±3.8)**	**70.5**	**(±3.8)**	**69.8**	**(±3.6)**
Arkansas	88.3	(±5.9)	74.3	(±8.3)	79.7	(±7.3)	35.8	(±7.9)	56.0	(±9.0)	57.1	(±8.9)
Louisiana	88.1	(±5.1)	78.5	(±6.4)	81.6	(±5.7)	50.4	(±7.7)	69.6	(±7.3)	69.1	(±7.5)
New Mexico	89.1	(±4.6)	79.8	(±6.4)	67.5	(±7.0)	49.3	(±7.5)	68.7	(±7.0)**	65.7	(±7.2)
Oklahoma	89.8	(±3.8)	79.2	(±5.4)	76.7	(±5.4)**	51.8	(±6.5)	58.8	(±6.4)	62.7	(±6.3)
Texas	92.7	(±2.8)	81.5	(±4.5)	81.8	(±3.9)**	64.2	(±5.3)**	73.8	(±5.2)	72.5	(±5.0)**
Bexar County	93.0	(±3.7)	79.4	(±6.5)	73.0	(±6.9)	64.3	(±7.2)	67.2	(±7.5)	70.6	(±7.1)
City of Houston	92.4	(±4.5)	85.0	(±6.3)	83.2	(±7.9)	65.8	(±9.0)	80.8	(±7.6)	77.6	(±7.4)
El Paso County	93.7	(±3.3)**	76.7	(±6.1)	74.5	(±6.2)	64.8	(±6.8)	70.6	(±6.7)	69.7	(±6.6)
Rest of state	92.7	(±3.5)	81.4	(±5.7)	82.7	(±4.8)**	63.9	(±6.7)	73.5	(±6.6)**	72.0	(±6.3)**
**HHS Region VII**	**91.1**	**(±2.7)**	**84.5**	**(±3.3)**	**79.1**	**(±3.5)**	**54.9**	**(±4.5)**	**73.5**	**(±4.0)**	**71.9**	**(±4.0)**
Iowa	94.5	(±3.9)	89.6	(±4.4)	79.5	(±7.2)**	57.5	(±8.6)	74.7	(±8.2)	78.3	(±6.7)
Kansas	89.4	(±4.7)	81.6	(±6.1)	77.2	(±6.5)	60.2	(±7.6)	72.6	(±6.9)**	68.7	(±7.1)
Missouri	89.8	(±5.3)	82.1	(±6.6)	79.2	(±6.3)	45.9	(±8.5)	72.4	(±7.5)	67.9	(±7.7)
Nebraska	92.5	(±4.1)	88.3	(±4.7)	81.3	(±5.3)	69.5	(±6.5)	76.2	(±6.2)	79.0	(±5.9)
**HHS Region VIII**	**89.2**	**(±2.7)**	**84.2**	**(±3.0)**	**70.4**	**(±3.7)**	**54.5**	**(±4.0)**	**74.1**	**(±3.5)**	**71.4**	**(±3.7)**
Colorado	86.0	(±5.5)	81.2	(±6.0)	60.2	(±7.3)	47.6	(±7.4)	73.8	(±6.6)	69.2	(±6.9)
Montana	87.3	(±5.2)	79.0	(±6.4)	73.9	(±6.8)	46.4	(±8.5)	65.5	(±8.2)	65.4	(±8.1)
North Dakota	91.4	(±3.8)	78.6	(±5.9)	82.0	(±5.9)	59.5	(±6.8)	78.4	(±5.4)	72.0	(±6.2)
South Dakota	93.1	(±4.4)	86.5	(±5.8)	70.9	(±7.8)	55.4	(±8.3)	68.7	(±7.8)	73.8	(±7.7)**
Utah	92.6	(±3.6)	90.3	(±4.1)**	81.2	(±5.5)	67.6	(±6.8)**	78.3	(±5.8)	75.2	(±6.1)
Wyoming	89.0	(±5.4)	80.9	(±6.6)	67.0	(±8.0)	33.6	(±7.6)	65.7	(±8.0)	70.0	(±7.7)
**HHS Region IX**	**90.8**	**(±4.2)**	**82.1**	**(±5.1)**	**71.9**	**(±6.1)**	**56.8**	**(±6.7)**	**75.1**	**(±5.7)**	**68.2**	**(±6.2)**
Arizona	91.4	(±3.7)	76.6	(±6.6)	79.1	(±5.8)	55.4	(±8.1)	70.9	(±7.5)	65.1	(±7.7)
California	90.7	(±5.3)	83.1	(±6.4)	70.3	(±7.7)	56.8	(±8.4)	76.8	(±7.2)	69.3	(±7.8)
Hawaii	92.8	(±3.8)	83.7	(±6.1)	77.3	(±6.7)	54.2	(±8.0)	73.3	(±6.9)	66.5	(±8.2)**
Nevada	90.4	(±3.5)	81.1	(±5.0)	75.4	(±5.6)	61.1	(±6.4)	62.1	(±6.5)	60.6	(±6.4)
**HHS Region X**	**91.9**	**(±2.5)** **	**81.2**	**(±4.1)**	**71.6**	**(±4.3)**	**56.2**	**(±5.0)**	**72.1**	**(±4.4)**	**69.2**	**(±4.6)**
Alaska	90.5	(±3.6)	75.5	(±6.1)	59.4	(±7.0)	52.5	(±7.2)	64.2	(±6.8)	63.9	(±6.8)
Idaho	91.1	(±4.3)	84.2	(±5.3)	72.7	(±6.5)	60.7	(±7.3)	74.6	(±6.4)	70.2	(±6.9)
Oregon	89.4	(±4.4)	83.8	(±5.2)	66.8	(±6.3)	55.9	(±6.7)	64.3	(±6.7)	66.6	(±6.5)
Washington	93.5	(±3.9)**	79.8	(±7.0)	75.0	(±7.1)	55.7	(±8.4)	76.3	(±7.3)	70.8	(±7.8)
*Range*	*(86.0–96.3)*		*(74.3–93.3)*		*(44.8–88.0)*		*(33.6–72.1)*		*(56.0–84.4)*		*(57.1 – 82.1)*	
**Territories**												
Guam	84.9	(±5.5)	71.5	(±7.2)	87.7	(±4.7)	45.8	(±7.5)	8.0	(±3.8)	50.3	(±7.8)
U.S. Virgin Islands	59.0	(±8.7)	51.1	(±8.8)	78.5	(±6.6)	18.6	(±6.5)	23.7	(±7.8)	39.8	(±8.5)

**Abbreviations:** MMR = measles, mumps, and rubella vaccine; DTaP = diphtheria, tetanus toxoids, and acellular pertussis vaccine (includes children who might have been vaccinated with, diphtheria and tetanus toxoids vaccine, or diphtheria, tetanus toxoids, and pertussis vaccine; HepB = hepatitis B vaccine; HepA = hepatitis A vaccine; CI = confidence interval; Hib = *Haemophilus influenzae* type b vaccine; PCV = pneumococcal conjugate vaccine.

* The combined (4:3:1:3*:3:1:4) vaccine series includes ≥4 doses of DTaP, ≥3 doses of poliovirus vaccine, ≥1 dose of measles-containing vaccine, full series of Hib vaccine (≥3 or ≥4 doses, depending on product type), ≥3 doses of HepB, ≥1 dose of varicella vaccine, and ≥4 doses of PCV.

† Children in the 2013 National Immunization Survey were born January 2010-May 2012.

§ HepB administered from birth through age 3 days.

¶ Either ≥2 or ≥3 doses of rotavirus vaccine, depending on product type received (≥2 doses for Rotarix [RV1] or ≥3 doses for RotaTeq [RV5]).

** Statistically significant increase in coverage compared with 2012 estimates from the National Immunization Survey (p<0.05)

## Footnotes

* Additional information is available on *Healthy People 2020* at http://healthypeople.gov/2020/topicsobjectives2020/objectiveslist.aspx?topicid=23.

† The *Healthy People 2020* target for the birth dose (day 0–3) of HepB is 85%, measured by annual birth cohort. In the three most recent completed birth cohorts measured by NIS, coverage with the birth dose of HepB was 65% for children born in 2008, 70.6% for children born in 2009, and 74.5% for children born in 2010.

§ All identified cell telephone households were eligible for interview. Sampling weights were adjusted to correct for dual-frame (landline and cell telephone) sampling, nonresponse, noncoverage, and overlapping samples of mixed (landline and cellular) telephone users. A description of NIS dual-frame survey methodology and its effect on reported vaccination estimates is available at http://www.cdc.gov/vaccines/imz-managers/coverage/nis/child/dual-frame-sampling.html.

¶ The local areas separately sampled for the 2013 NIS included areas that receive federal Section 317 immunization funds and are included in the NIS sample every year (Chicago, Illinois; New York, New York; Philadelphia County, Pennsylvania; Bexar County, Texas; and Houston, Texas) and one additional sampled area (El Paso County, Texas). The 2013 NIS was also conducted in USVI and Guam, but these areas were excluded from national coverage estimates.

** A description of the statistical methodology of the NIS is available at ftp://ftp.cdc.gov/pub/health_statistics/nchs/dataset_documentation/nis/nispuf12_dug.pdf.

†† Children from USVI (n = 201) and Guam (n = 248) were excluded from the national estimates. For completed interviews, for Guam, 63 by landline (64.3%) and 185 by cell phone (63.6%) had adequate provider data. For completed interviews, for USVI, 55 by landline (55.9%) and 146 by cell phone (49.8%) had adequate provider data.

§§ The CASRO household response rate, calculated as the product of the resolution rate (percentage of the total telephone numbers called that were classified as nonworking, nonresidential, or residential), screening completion rate (percentage of known households that were successfully screened for the presence of age-eligible children), and the interview completion rate (percentage of households with one or more age-eligible children that completed the household survey). For USVI, the landline and cell phone sample CASRO rates were 72.8% and 37.2%, respectively. For Guam, the landline and cell phone sample CASRO rates were 54.6% and 29.7%, respectively. Additional information is available at http://www.casro.org. The CASRO response rate is equivalent to the American Association for Public Opinion Research (AAPOR) type 3 response rate. Information about AAPOR response rates is available at http://www.aapor.org/am/template.cfm?section=standard_definitions1&template+/cm/contentdisplay.cfm&contented=1814.

¶¶ Coverage for primary Hib series was based on receipt of ≥2 or ≥3 doses, depending on product type received. The PRP-OMB Hib products require a 2-dose primary series with doses at ages 2 months and 4 months. All other Hib products require 3-dose primary series with doses at ages 2, 4, and 6 months. Coverage for the full series, which includes the primary series and a booster dose, was based on receipt of ≥3 or ≥4 doses, depending on product type received. All Hib products require a booster dose at age 12–15 months.

*** Coverage for rotavirus vaccine was based on ≥2 or ≥3 doses, depending on product type received (≥2 doses for Rotarix [RV1], licensed in April 2008, and ≥3 doses for RotaTeq [RV5], licensed in February 2006). ACIP does not recommend using the two rotavirus vaccines interchangeably, but in the event that using more than one product cannot be avoided because of nonavailability of vaccine used to initiate series, then a total of 3 doses are required if RV5 is one of the vaccine doses (e.g., acceptable mixed series could be RV1-RV5-RV5/RV1-RV5-RV1/RV5-RV1-RV1/RV5-RV1-RV5/RV5-RV5-RV1). Additional information at http://www.cdc.gov/mmwr/preview/mmwrhtml/rr5802a1.htm.

††† The combined (4:3:1:3*:3:1:4) vaccine series includes ≥4 doses of DTaP/diphtheria and tetanus toxoids vaccine/diphtheria, tetanus toxoids, and pertussis vaccine, ≥3 doses of poliovirus vaccine, ≥1 dose of measles-containing vaccine, ≥3 or ≥4 doses of Hib (depending on product type of vaccine), ≥3 doses of HepB, ≥1 dose of varicella vaccine, and ≥4 doses of PCV.

§§§ Poverty level uses income and family size to categorize households into 1) at or above the poverty level and 2) below the poverty level. Poverty level was based on 2011 U.S. Census poverty thresholds, available at http://www.census.gov/hhes/www/poverty/data/threshld.

¶¶¶ Child’s race/ethnicity was reported by their parent or guardian. Children categorized in this report as white, black, Asian, American Indian/Alaska Native, or multiracial were identified as non-Hispanic by their parent or guardian. Children identified as multiracial had more than one race category selected. Persons identified as Hispanic might be of any race.

**** Additional information available at http://www.amstat.org/meetings/jsm/2012/onlineprogram/abstractdetails.cfm?abstractid=304324.
